# Quantifying the U.S. surgical instrument fleet and its reprocessing burden: implications for cost and sustainability in healthcare

**DOI:** 10.1186/s12913-026-14663-3

**Published:** 2026-04-30

**Authors:** Abner Mácola, William Estes, Peter F. Nichol

**Affiliations:** 1 Department of Surgery, University of Wisconsin School of Medicine, and Public Health, H4/746 CSC 600 Highland Ave, Madison, WI 53792 USA; 2 Ascendco Health, 350 N Orleans St, Chicago, IL 60654 USA

**Keywords:** Surgical instruments, Reprocessing, National fleet sample, Informatics, Waste

## Abstract

**Background:**

Reusable surgical instruments require repeated sterilization between procedures, yet many instruments prepared for surgery are never used. The national scale of the reusable surgical instrument fleet and its associated reprocessing burden remain poorly quantified.

**Methods:**

Aggregated operational data from 251 U.S. healthcare facilities encompassing 2,618 operating rooms were analyzed to quantify instrument inventories, sterilization volume, and instrument loss. National estimates were derived by scaling operating room–level metrics to reported U.S. operating room capacity. Published sterilization cost estimates and instrument utilization rates were applied to estimate national sterilization expenditures and idle reprocessing costs. Replacement costs for missing instruments were modeled using log normal price distributions and Monte Carlo simulation.

**Results:**

The dataset included 336,784 trays and 6.7 million instruments, with 236 million instruments reprocessed annually. National extrapolation suggests a reusable instrument fleet of approximately 112 million instruments and nearly 4 billion annual reprocessing events. Estimated annual sterilization costs ranged from $1.3 to $11.8 billion.

**Conclusions:**

This study provides an initial estimate of the national cost of surgical instrument reprocessing in the U.S. Further work is required to determine a national estimate of the percentage of instruments that go unused. Nevertheless, the scale of expenditures on this process indicates there will be significant opportunities to improve perioperative efficiency and reduce waste through better inventory management yielding substantial cost savings and decreasing the environmental footprint of surgical care.

## Introduction

Reusable surgical instruments form the backbone of operative care in all hospital operating rooms in the United States. To ensure sterility and regulatory compliance, instruments are routinely cleaned, assembled, packaged, and sterilized between cases. However, a growing body of empirical literature has demonstrated that a substantial proportion of instruments included in surgical trays are never used during the procedures for which they are prepared. Across multiple specialties, direct observational studies have reported instrument utilization rates as low as 13–22%, implying that the majority of instruments opened and sterilized for surgery remain unused [1–3].

This inefficiency has important economic implications. Sterilization and reprocessing include costs related to labor, utilities, consumables, equipment depreciation, quality assurance, and logistics. When unused instruments are repeatedly reprocessed, these costs accumulate without contributing to patient care. In parallel, surgical instruments are routinely lost, misplaced, damaged, or retired, generating additional replacement costs and increasing the effective size and complexity of hospital instrument inventories.

Despite extensive single-institution and specialty-specific studies on tray optimization and instrument utilization, the national-scale economic burden of the reusable surgical instrument fleet remains poorly characterized. In particular, no prior work has combined large-scale operational data with national extrapolation to estimate the magnitude of sterilization volume, idle reprocessing, and instrument loss across the U.S. healthcare system [2, 4].

In this study, we use aggregated data from 251 U.S. healthcare facilities and 2,618 operation rooms (ORs) to quantify reprocessing volume, inventory composition, and instrument loss. We then extrapolate these findings to the national level using publicly available estimates of U.S. operating room capacity. By integrating published estimates of reprocessing cost and instrument utilization, we estimate the annual national cost attributable to sterilization, idle reprocessing, and missing instruments, and we characterize the degree of concentration in reprocessing burden across the instrument fleet.

To our knowledge, no prior study has estimated the size of the total U.S. reusable surgical instrument fleet using multi-facility operational data and national extrapolation. By quantifying both national fleet size and reprocessing volume, our work provides a benchmark for future interventions aimed at reducing idle sterilization, instrument loss, and the environmental footprint of perioperative care.

## Methods

### Data source and study cohort

We analyzed aggregated operational data provided by Ascendco Health, a Chicago-based healthcare technology firm company managing surgical instrument inventories and sterile processing operations across 251 U.S. healthcare facilities and 2,618 ORs as of March 2026. The data contained facility and operating room level summaries of active surgical trays, active instruments, total instruments reprocessed during the preceding 365 days, trays containing missing instruments, and counts of missing instruments. All data were deidentified and aggregated at the facility or operating room level, and no patient level or procedure level information was included.

Facilities included in the dataset were geographically distributed across 40 U.S. states and represented a wide range of healthcare markets and facility sizes. Participating institutions were located in the Northeast, Midwest, South, and Western United States and included both large metropolitan hospital systems and smaller regional hospitals. The dataset primarily includes hospital-based operating rooms. Ambulatory surgical centers were not systematically represented in the dataset and therefore were not included in national extrapolation.

The dataset records inventory items rather than individual instruments. An inventory item may represent a single reusable instrument such as a scope, camera, or powered device, or it may represent a multi-instrument surgical set containing numerous instruments assembled for a specific procedure type. For multi-instrument sets, the dataset includes the number of instruments contained within each set. Total instrument counts were therefore calculated by multiplying the number of sterilization cycles associated with each inventory item by the number of instruments represented by that item. This approach captures the true sterilization workload generated by each inventory item regardless of whether it represents a single instrument or a multi-instrument set.

### National operating room and hospital counts

National extrapolation was performed using estimates of U.S. surgical capacity from publicly available sources. The number of U.S. hospitals (6,093) was obtained from the American Hospital Association Annual Survey. Estimates of national operating room capacity were drawn from industry and health-system analyses reporting approximately 43,800 hospital-based operating rooms in the United States. Operating room–based scaling was used as the primary extrapolation approach, as sterilization volume and instrument inventory scale more directly with OR capacity than with hospital count [5].

### Extrapolation of reprocessing volume and inventory

Mean values for instruments reprocessed per OR per year, active instruments per OR, and trays per OR were calculated from the company dataset. National totals were estimated by multiplying these per-OR means by the estimated number of U.S. operating rooms. Parallel hospital-based extrapolations were performed as sensitivity analyses. In addition to estimating national reprocessing volume, we present a national estimate of the total U.S. reusable surgical instrument fleet derived from observed per-OR inventory levels, which has not been previously quantified at scale in the peer-reviewed literature.

### Instrument utilization and idle sterilization

Instrument utilization fractions were derived from prior peer-reviewed studies reporting the proportion of instruments used intraoperatively among those opened and sterilized. Across studies, reported utilization rates ranged from approximately 13% to 30%, corresponding to unused fractions of 70–87% [3]. Idle sterilization was defined as the reprocessing of instruments opened during procedures but not ultimately used. National idle reprocessing volume was estimated by applying these unused fractions to the extrapolated national reprocessing volume.

### Cost assumptions

Per-instrument reprocessing costs were derived from published estimates in the literature, which report values ranging from approximately $0.34 to over $3.00 per instrument when accounting for labor, supplies, utilities, and depreciation. Three cost scenarios were modeled to reflect this range. A low cost estimate of 0.34 dollars per instrument, a benchmark mid-range estimate of 0.77 dollars per instrument, and a high cost estimate of 3.00 dollars per instrument were applied to the estimated national reprocessing volume to generate corresponding national cost estimates [3, 6, 7].

Replacement costs for missing instruments were estimated using instrument price distributions derived from the observed composition of the instrument inventory. Inventory items were categorized into major equipment groups including cameras, scopes, powered equipment, surgical sets, and instrument packs. Observed mean prices and standard deviations for each category were used to parameterize log normal price distributions, reflecting the right skewed distribution typical of medical equipment pricing.

Uncertainty in instrument acquisition costs was evaluated using probabilistic sensitivity analysis. Monte Carlo simulation was used to generate 10,000 draws from the category specific log normal price distributions. In each simulation, instrument prices were sampled according to the observed composition of the inventory and weighted by the relative frequency of each instrument category. The resulting distribution of simulated prices was used to estimate the mean replacement cost per instrument and the corresponding national replacement cost associated with missing instruments.

In addition to the probabilistic analysis, deterministic cost scenarios based on weighted quartiles of the price distribution were reported to provide conservative low, mid, and high estimates of replacement cost.

### Inequality and concentration analysis

The distribution of sterilization workload across inventory items was evaluated using Pareto analysis and the Gini coefficient. Sterilization workload for each inventory item was calculated as the product of annual sterilization frequency and the number of instruments represented by that item. This approach accounts for the heterogeneous structure of the inventory in which some items represent single instruments and others represent multi-instrument surgical sets.

Inventory items were ranked according to their total annualized contribution to sterilization workload. Cumulative contribution to total sterilization volume was calculated, and the proportion of inventory items accounting for 80% of total sterilization activity was identified. The Gini coefficient was calculated to quantify the degree of concentration in sterilization workload across inventory items.

### Statistical analysis

The analytic dataset included 2,618 operating rooms, representing approximately 6% of the estimated 43,800 hospital-based operating rooms in the United States. Given this sample size, national estimates derived from per–operating room averages are statistically precise. Even under conservative assumptions of high inter-facility variability (coefficient of variation ≈ 1.0), the resulting 95% confidence intervals around mean estimates are within approximately ± 4%. Finite population correction had minimal impact due to the large population size relative to the sample. Accordingly, uncertainty in national extrapolations is driven primarily by structural representativeness rather than sample size. All analyses were conducted using R statistical software version 4.3.3 within the RStudio integrated development environment.

## Limitations

This study has several limitations. First, the analytic cohort represents a large operational sample of U.S. operating rooms but does not constitute a probability sample, as inclusion was determined by participation in a commercial sterile processing management platform rather than random selection. Although participating facilities were geographically distributed across 40 states and included institutions of varying sizes and healthcare markets, national extrapolations assume that observed operating room level inventory and reprocessing patterns are broadly representative of U.S. hospitals. Differences in local practice patterns, staffing models, or sterilization workflows could influence instrument utilization and reprocessing volumes and may limit the generalizability of national estimates.

Second, the dataset captures sterilization volumes and instrument inventories but does not directly measure instrument utilization during individual surgical procedures. Idle reprocessing volumes were therefore estimated using utilization fractions reported in prior peer-reviewed studies rather than directly measured utilization data. While these estimates are consistent with the surgical tray utilization literature, actual utilization rates may vary by surgical specialty, procedure type, and institutional practice patterns.

Third, replacement costs for missing instruments were estimated using modeled price distributions rather than institution specific purchasing data. Although probabilistic sensitivity analysis using log normal price distributions was performed to capture uncertainty in instrument acquisition costs, actual replacement costs may vary depending on vendor contracts, purchasing agreements, and equipment specifications at individual hospitals.

Fourth, the analysis relies on aggregated operational data and does not link individual instruments to specific procedures, surgeons, or surgical specialties. As a result, the study identifies inventory items that contribute most to sterilization workload but cannot directly determine which instruments within those sets are most frequently unused during procedures.

Finally, the dataset captures instrument inventories and reprocessing volumes but does not directly measure downstream operational consequences such as surgical delays, case cancellations, or additional labor costs associated with locating or replacing missing instruments. The estimated economic burden therefore likely underestimates the broader operational impact of inefficiencies in surgical instrument management.

Despite these limitations, the large scale of the dataset, the geographic diversity of participating facilities, and the consistency of observed concentration patterns across inventory items support the robustness of the principal findings.

## Results

### Facility- and OR-level characteristics

Across the analytic cohort, the dataset included 336,784 active trays and 6,707,325 active instruments across 2,618 operating rooms. Over the period, a total of 236,060,012 instruments were reprocessed. Missing instruments were identified in 25,240 trays, with a total of 87,050 missing instruments recorded across those trays. This corresponds to a tray level missing instrument rate of 7.5%. Additional descriptive characteristics of the dataset are summarized in Table 1.

**Table 1:** Descriptive characteristics of the analytic dataset. Summary of facility- and operating room–level characteristics derived from the aggregated operational dataset

Metric	Value
Healthcare facilities	251
Operating rooms	2,618
U.S. states represented	40
Active trays	336,784
Active instruments	6,707,325
Instruments reprocessed (365 days)	236,060,012
Trays with missing instruments	25,240
Missing instruments	87,050
Tray-level missing-instrument rate	7.5%

### Concentration of sterilization burden

**Fig. 1:**
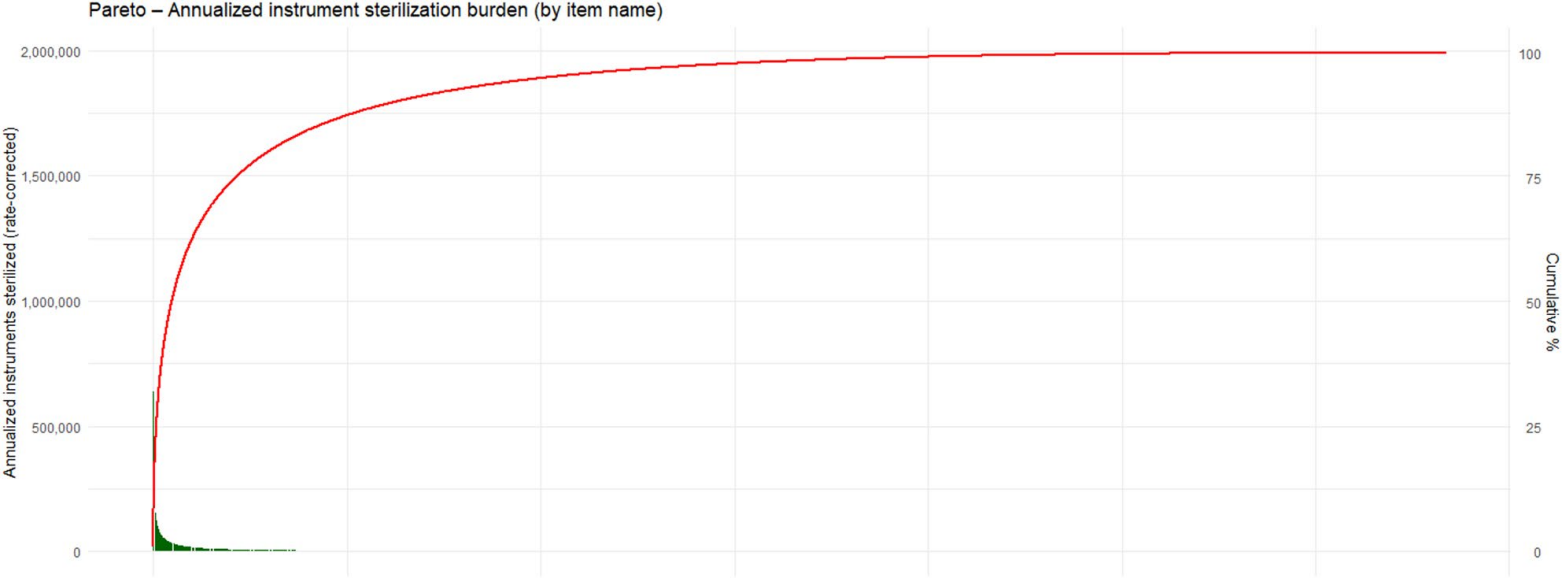
Pareto distribution of annualized surgical instrument sterilization burden by inventory item. Bars represent the annualized number of instruments sterilized for each inventory item, accounting for the number of instruments contained within that item. Items are ranked by total sterilization volume. The red curve shows the cumulative percentage of total sterilization activity, illustrating the strong concentration of sterilization workload among a small subset of inventory items

Sterilization workload was highly concentrated across inventory items. Pareto analysis demonstrated that approximately 12.1% of inventory items accounted for 80% of total annualized sterilization volume. The remaining 87.9% of items contributed only 20% of sterilization activity. The Gini coefficient for annualized instrument reprocessing burden was 0.84, indicating a high degree of concentration in sterilization workload (Fig. 1.).

The largest contributors to sterilization workload were commonly used surgical sets that contain large numbers of instruments and are repeatedly processed across procedures. The highest contributing inventory items included hand surgery sets, minor procedure sets, general minor sets, dilation and curettage sets, cesarean section sets, basic orthopedic sets, and minor orthopedic sets. Because these sets contain dozens of instruments and are frequently used across multiple surgical services, they account for a disproportionate share of total sterilization activity.

### National extrapolation of reprocessing volume

Scaling the observed operating room level inventory and sterilization volumes to the estimated 43,800 hospital based operating rooms in the United States yielded a national reusable surgical instrument inventory of approximately 112,215,750 instruments. Annual sterilization volume was estimated at approximately 3,949,361,545 instrument reprocessing events per year. A summary of national and per–operating room estimates of instrument inventory, reprocessing volume, and associated costs is presented in Table 2.

**Table 2:** National and per–operating room estimates of surgical instrument fleet and reprocessing costs (US dollars)

Metric	Per OR (Annual)	National Estimate (U.S.)
Active instruments (fleet size)	2,561	112,215,750
Active trays	129	5,629,000
Instruments reprocessed	90,200	3,949,361,545
Missing instruments	33	1,456,375
Sterilization cost ($0.34/instrument)	$30,700	$1.34 billion
Sterilization cost ($0.77/instrument)	$69,500	$3.04 billion
Sterilization cost ($3.00/instrument)	$270,600	$11.8 billion

Estimates by scaling trays per OR to 43,8000Rs

This corresponds to an average of approximately 77 instruments reprocessed per surgical procedure, assuming approximately 51 million surgical procedures annually nationwide [8].

The extrapolated national estimate of trays containing missing instruments was approximately 422,273 trays, corresponding to approximately 1,456,375 missing instruments annually across U.S. hospitals.

### National sterilization cost and idle reprocessing

Applying per instrument sterilization cost estimates to the extrapolated national reprocessing volume yielded substantial national cost estimates. At a cost of $0.34 per instrument, annual sterilization expenditures were estimated at approximately $1.34 billion. Using a benchmark estimate of $0.77 per instrument, the corresponding annual cost increased to approximately $3.04 billion. Under higher cost assumptions of $3.00 per instrument, national sterilization expenditures exceeded $11.8 billion annually. When previously reported instrument utilization fractions were applied, indicating that 70% to 87% of sterilized instruments are not ultimately used during procedures, a large proportion of these costs corresponded to idle reprocessing. Under these assumptions, the annual cost associated with reprocessing unused instruments was estimated at approximately $0.94 billion under the lowest cost scenario, more than $2.4 billion under the benchmark cost assumption, and over $10 billion annually under the highest cost scenario.

### Replacement cost of missing instruments

**Fig. 2:**
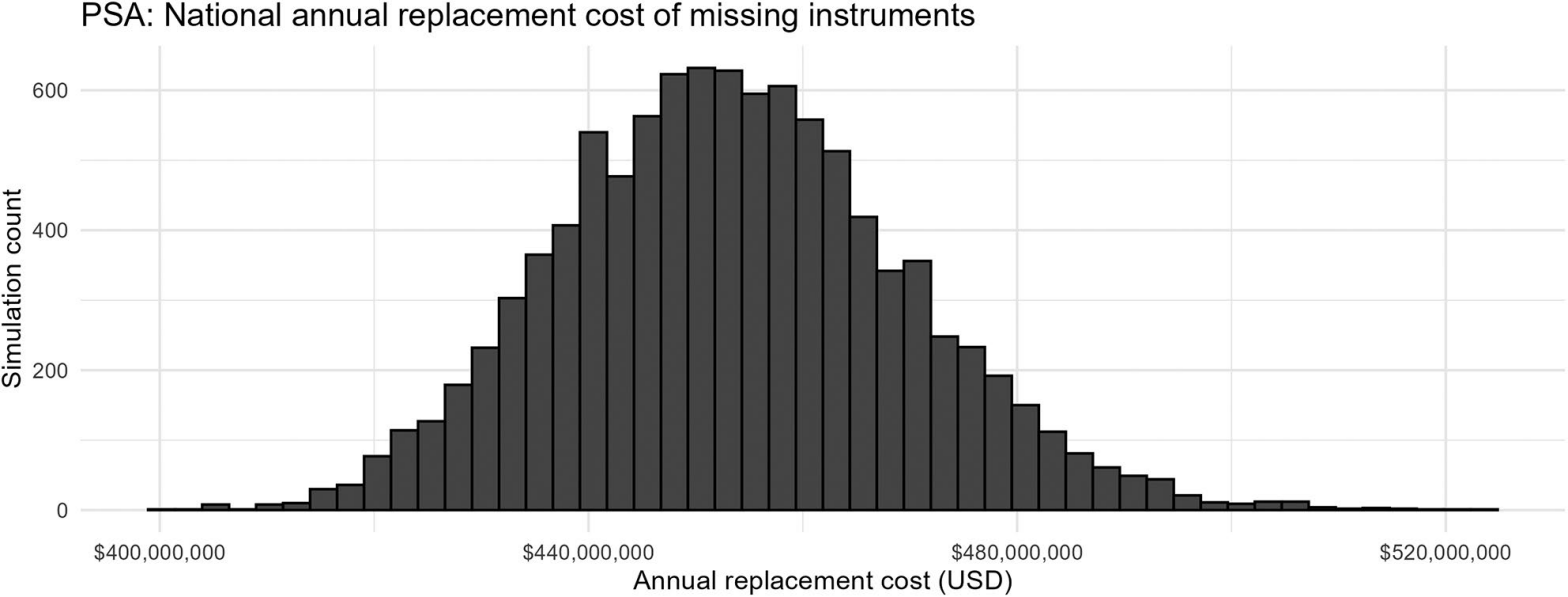
Monte Carlo simulation of national annual replacement costs for missing surgical instruments**.** Distribution of national replacement cost estimates generated from 10,000 Monte Carlo simulations using log normal price distributions derived from instrument categories. The histogram illustrates the uncertainty range of national annual replacement costs associated with missing instruments

Deterministic replacement cost scenarios were used to estimate the national economic impact of missing instruments. Using the weighted first quartile of instrument price distributions, the estimated national replacement cost was approximately $80.6 million annually. When the weighted median instrument price was applied, the estimated annual replacement cost increased to approximately $317.4 million. Using the weighted third quartile of the price distribution yielded an estimated annual replacement cost of approximately $643.9 million. Probabilistic sensitivity analysis using Monte Carlo simulation produced similar results. The simulated national replacement cost distribution yielded a 95% uncertainty interval ranging from approximately $423.3 million to $486.4 million annually (Fig. 2.).

## Discussion

Hospitals operate under persistent financial strain, with many organizations facing thin or negative operating margins that are sensitive to payer mix and uncompensated care [9]. Large national analyses have shown that safety-net intensity and uncompensated care are associated with lower operating margins, reinforcing how small efficiency gains in perioperative operations can materially affect sustainability [10]. In parallel, Medicare policy changes and payment structures continue to shape hospital finances; for example, elimination of specific Medicare reimbursement components has been modeled to reduce margins further for a subset of hospitals, highlighting how limited financial slack constrains the ability to absorb avoidable costs [11, 12].

These pressures are likely to intensify as U.S. surgical demand grows with population aging. Longstanding projections indicate substantial increases in demand for surgical services driven by demographic shifts [13]. Procedure-specific forecasts show continued growth in high-volume specialties such as total joint arthroplasty, and oncologic care needs are expected to expand as cancer burden becomes increasingly concentrated among older adults [14, 15]. The convergence of rising procedural volume and constrained reimbursement heightens the importance of reducing work that does not contribute to patient care within routine perioperative workflows.

Within this context, sterile processing and surgical instrumentation represent a particularly actionable target for operational improvement. Our findings show that a small subset of inventory drives most sterilization workload, and corroborates the broader literature demonstrating that instrument sets frequently include substantial redundancy and that tray optimization can reduce reprocessing volume without compromising operative function [16]. Conceptually, these results support a shift from “sterilize-by-default” toward “sterilize-for-need,” using data-driven standardization, peel-packing for low-probability items, and continuous set governance to reduce repeated handling of instruments that rarely contribute to patient care.

Reducing waste in reprocessing also intersects directly with workforce sustainability. Sterile processing departments (SPDs) are labor-intensive systems that depend on trained technicians, inspection workflows, and high-reliability execution. A recent national survey documents economic challenges faced by SPD workers in the United States, providing timely evidence that labor and retention pressures are part of the operational reality in which hospitals must implement any efficiency strategy [9]. In this setting, reducing unnecessary reprocessing can be framed not only as cost containment, but also as a way to decrease avoidable workload, mitigate burnout risk, and preserve capacity for quality-critical tasks.

Beyond the economic implications, inefficiencies in surgical instrument reprocessing have important sustainability consequences. Operating rooms are recognized as major contributors to healthcare-related greenhouse gas emissions, and prior life-cycle assessments have demonstrated that perioperative activities, including instrument decontamination, packaging, and steam sterilization, consume substantial energy and water resources. Studies have shown that reprocessing workflows and packaging choices materially influence the environmental footprint of surgical care, and that reducing unnecessary instrument handling can lower both carbon and water intensity without compromising clinical performance [17–19]. In this context, the high concentration of reprocessing burden observed in the present analysis suggests that targeted inventory rationalization could yield environmental benefits that parallel the financial savings, reinforcing the concept of a dual economic and environmental dividend from waste reduction in sterile processing [19].

Future work should prioritize scalable, technology-enabled approaches to translate utilization measurement into sustained operational improvement. Instrument tracking systems using barcode or radio-frequency identification have demonstrated the feasibility of continuous monitoring of instrument flow and loss, enabling data-driven decisions regarding tray composition and instrument retirement [20]. In parallel, recent advances in computer vision and artificial intelligence offer promising opportunities to automate instrument recognition, counting, and utilization assessment, reducing reliance on manual documentation and mitigating known vulnerabilities in human-based informatics within operating room and sterile processing workflows [21, 22]. Integrating these technologies with procedure-specific demand modeling could support adaptive, evidence-based tray design that aligns sterilization effort with actual clinical need. Additionally, optimization of containment, transport, and packaging systems, such as standardized rigid container workflows, should be evaluated as complementary interventions to reduce handling errors, environmental impact, and reprocessing burden [23]. Together, these approaches represent a clear pathway for future research aimed at improving the sustainability, resilience, and efficiency of surgical instrument management at scale.

## Conclusion

Using a large multi-facility operational dataset and national extrapolation, this study provides the first data-driven estimates of the size of the U.S. reusable surgical instrument fleet and its associated annual reprocessing burden. The findings demonstrate that surgical instrument sterilization is highly concentrated, with a small fraction of the inventory accounting for the majority of reprocessing activity, and that substantial volumes of sterilization likely occur without contributing to patient care. These patterns indicate that meaningful reductions in cost, operational burden, and environmental impact can be achieved by targeting a limited subset of instruments rather than undertaking system-wide changes. As healthcare systems face rising procedural demand, constrained reimbursement, and increasing sustainability pressures, data-driven optimization of surgical instrument inventories represents a practical and scalable opportunity to improve efficiency and resilience in perioperative care.

## Data Availability

Data will be provided by the authors upon reasonable request.

## Abbreviations

SPD: Sterile processing department
OR: Operating room
